# Directional charge delocalization dynamics in semiconducting 2H-MoS$_{2}$ and metallic 1T-Li$_{\mathrm{x}}$MoS$_{2}$

**DOI:** 10.1038/s41598-021-86364-2

**Published:** 2021-03-25

**Authors:** Robert Haverkamp, Nomi L. A. N. Sorgenfrei, Erika Giangrisostomi, Stefan Neppl, Danilo Kühn, Alexander Föhlisch

**Affiliations:** 1 Methods and Instrumentation for Synchrotron Radiation Research PS-ISRR, Helmholtz-Zentrum Berlin für Materialien und Energie GmbH, Albert-Einstein-Straße 15, 12489 Berlin, Germany; 2 Institut für Physik und Astronomie, Universität Potsdam, Karl-Liebknecht-Straße 24/25, 14476 Potsdam, Germany

**Keywords:** Physics, Materials science

## Abstract

The layered dichalcogenide MoS$_{2}$ is relevant for electrochemical Li adsorption/intercalation, in the course of which the material undergoes a concomitant structural phase transition from semiconducting 2H-MoS$_{2}$ to metallic 1T-Li$_{\mathrm{x}}$MoS$_{2}$. With the core hole clock approach at the S L$_{1}$ X-ray absorption edge we quantify the ultrafast directional charge transfer of excited S3p electrons in-plane ($\parallel$) and out-of-plane ($\perp$) for 2H-MoS$_{2}$ as $\tau _{2H,\parallel } = 0.38 \pm 0.08$ fs and $\tau _{2H,\perp } = 0.33 \pm 0.06$ fs and for 1T-Li$_{\mathrm{x}}$MoS$_{2}$ as $\tau _{1T,\parallel } = 0.32 \pm 0.12$ fs and $\tau _{1T,\perp } = 0.09 \pm 0.07$ fs. The isotropic charge delocalization of S3p electrons in the semiconducting 2H phase within the S-Mo-S sheets is assigned to the specific symmetry of the Mo-S bonding arrangement. Formation of 1T-Li$_{\mathrm{x}}$MoS$_{2}$ by lithiation accelerates the in-plane charge transfer by a factor of $\sim 1.2$ due to electron injection to the Mo-S covalent bonds and concomitant structural repositioning of S atoms within the S-Mo-S sheets. For excitation into out-of-plane orbitals, an accelerated charge transfer by a factor of $\sim 3.7$ upon lithiation occurs due to S-Li coupling.

## Introduction

Layered quasi-2D transition metal dichalcogenides (TMDs) are highly anisotropic crystals^1–3^ that are attractive for numerous fields of applications due to their unique inherent properties. Whereas the intralayer interaction is characterized by strong covalent bonding in these materials, the interlayer coupling by weak van der Waals (vdW) forces make layered TMDs amenable to intercalation of foreign atoms and molecules. In particular, molybdenum disulfide (MoS$_{2}$) based devices span the fields of photovoltaics^4^, hydrogen production^5,6^, electronics^7^, optoelectronics^8^ and even extend to the implementation of MoS$_{2}$ in future memresistive circuits and related applications^9,10^. Extraordinary capabilities have been demonstrated for MoS$_{2}$ based electrodes in rechargeable, high capacity lithium ion batteries (LIBs)^11^ as well as high energy and power density supercapacitors^12^ based on the efficient Li$^{+}$ intercalation.

MoS$_{2}$ possesses a repeated structure of S-Mo-S sheets occurring in distinct symmetries. The two most important polytypes are the naturally occurring 2H and the 1T allotropes^13,14^. While 2H-MoS$_{2}$ is a semiconductor with a layer dependent bandgap that increases from about 1.2 eV in the bulk up to about 1.9 eV for a monolayer^15,16^, 1T-MoS$_{2}$ is metallic with the Fermi level within the Mo d-band^17^. On a structural level, the transition from the hexagonal 2H phase with vertically aligned S atoms to the trigonal 1T structure corresponds to a collective shear displacement of S atoms equivalent to a $60^\circ$ rotation of one plane of S atoms^17^.

Both, experimental and computational studies show that the 2H-semiconductor to 1T-metal phase transition can be induced by electron injection into the 2H-MoS$_{2}$ crystal. Upon this charge doping, partial population of the Mo4d orbitals by an extra electron destabilizes the 2H crystal structure and leads to the transition into the 1T phase^14,18,19^. Experimentally this charge injection has been realized by electron microscopy^20,21^, laser excitation of deposited plasmonic nanoparticles^22^ and by alkali metal intercalation^14,23^, where Li atoms are the most efficient and commonly applied dopants.

Li with its 1s$^{2}$2s$^{1}$ electronic configuration can inject a 2s electron into the conduction band minimum of the 2H-MoS$_{2}$ crystal as the Li$^{+}$ cation is adsorbed and/or intercalated, leading to the formation of 1T-Li$_{\mathrm{x}}$MoS$_{2}$^14^. It has been demonstrated that, once MoS$_{2}$ based electrodes are loaded with Li atoms, 1T-Li$_{\mathrm{x}}$MoS$_{2}$ is not reversed back into 2H-MoS$_{2}$ anymore, but remains in the 1T phase. Thus loading cycles are not limited by structural fatigue from repeated volumetric changes between the 2H and 1T structure^12^.

An essential property that influences the performance of e.g. LIBs and supercapacitors is the electron mobility. In this context, our work provides access to the in- and out-of-plane electron transfer dynamics in 2H-MoS$_{2}$ and 1T-Li$_{\mathrm{x}}$MoS$_{2}$, relevant for electrochemical Li loading of MoS$_{2}$ based electrode materials, on the atomic level. Here the orbital specificity and sub-fs capabilities of the core-hole clock (CHC) method^24,25^ are essential since previously applied laser-based pump-probe investigations on MoS$_{2}$ and related heterostructures^26,27^ are limited on the few tens of fs timescale and lack of ability to provide directional selectivity. The CHC method has already been successfully applied to determine charge transfer dynamics in TaS$_{2}$^28^ and different MoS$_{2}$ based heterojunctions^29–31^, demonstrating its capability for measuring the directional charge transfer in layered TMDs. The basic principle of the CHC spectroscopy is elucidated in the methods section.

**Figure 1:**
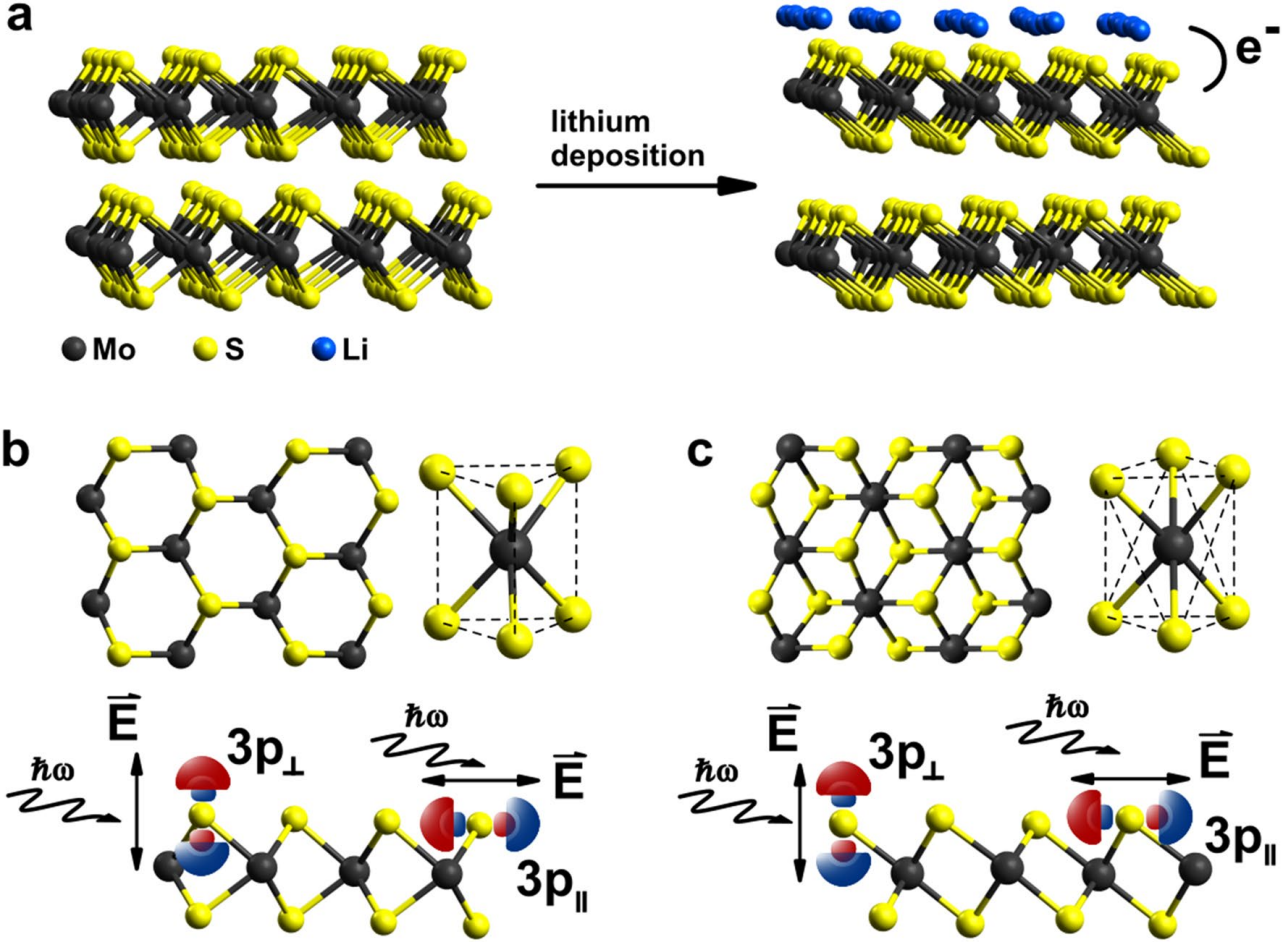
Structure of the semiconducting 2H and the metallic 1T phase of MoS$_{2}$ and the directional excitation of an electron into unoccupied S3p states following absorption of linearly polarized X-rays at the S L$_{1}$-edge in the CHC experiment. (**a**) Lithium deposition onto 2H-MoS$_{2}$ leads to concomitant intercalation/adsorption of Li$^{+}$ cations and electron injection, causing the phase transition and formation of 1T-Li$_{\mathrm{x}}$MoS$_{2}$. (**b**, **c**) Crystallographic structures in top- and side-views of a monolayer of the 2H and the 1T phase. CHC spectroscopy prepares selectively in-plane ($\parallel$) and out-of-plane ($\perp$) S3p excited states. Black, yellow and blue spheres represent Mo, S and Li atoms, respectively. The crystallographic structure was build using Avogadro software version 1.2.0^32^ and edited with Inkscape software version 0.92.3 (www.inkscape.org).

## Results and discussion

Figure 1 summarizes the essence of the structural properties of 2H-MoS$_{2}$ and 1T-Li$_{\mathrm{x}}$MoS$_{2}$ as well as the preparation of 1T-Li$_{\mathrm{x}}$MoS$_{2}$ through electron injection following Li adsorption and/or intercalation. Finally it also shows how the CHC method allows to detect in-plane and out-of-plane charge transfer times of an electron excited into S3p in-plane and out-of-plane orbital states, respectively. Figure 1a depicts schematically how *in situ* Li deposition directly onto the clean 2H-MoS$_{2}$ surface leads to the 1T phase, following the ultra-high vacuum (UHV) compatible preparation described by Papageorgopoulos et al^23^, with further details given in the methods section. This UHV compatible approach has shown to lead to the same 1T-Li$_{\mathrm{x}}$MoS$_{2}$ phase equivalently as do methods involving alkali solution^5,12^ or electrochemical preparations^13,14^. It has been shown that the 1T phase, prepared by lithiation of MoS$_{2}$, stabilizes locally though lattice distortions by forming the so-called 1T’ structure which results in the coexistence of the 1T and the distorted 1T’ crystallographic structure within the MoS$_{2}$ lattice^33^. The differentiation between the 1T/1T’ and 2H phase has been verified independently by techniques like Raman-^4–6,22,34^, photoluminescence-^22,35^, X-ray photoelectron spectroscopy (XPS)^4,6,35^, high-resolution electron microscopy^4,21^ , nanomechanics^36^ and can also be accomplished by valence band photo emission^37^. While laser based optical methods like Raman spectroscopy as well as electron microscopy are suitable techniques to study the crystallographic phase and therefore allow the unambiguous determination of the 1T and 1T’ structure in MoS$_{2}$, it has been shown that both methods depending on the laser power^38^, respectively the electron dose^21^, influence the crystallographic phase. We confirmed the successful phase transition upon lithiation by *in situ* XPS of the valence band as well as the established S2p and Mo3d core level binding energies^23^. XPS offers the advantage that it allows to detect the 2H to 1T/1T’ phase transition as well as the phase ratio by means of the S2p and Mo3d core level binding energies while valence band measurements clearly reveal the semiconductor to metal transition. However, it does not allow to distinguish the 1T and 1T’ structure. For that reason, hereinafter 1T will implicate both the undistorted and the distorted crystallographic phase. In our experiments, we achieved for our lithiated MoS$_{2}$ crystal a 1T phase concentration of 86 ± 5 % in the detection volume (quantitative fitting routine in the supplementary information).

In Fig. 1b,c we show the detailed structural properties within each S-Mo-S sheet for MoS$_{2}$ in the 2H (Fig. 1b) and 1T (Fig. 1c) phase. We also illustrated a representation of the directional selectivity based on the resonant excitation of an S2s core electron into the unoccupied S3p$_\parallel$ or S3p$_\perp$ conduction band states following the absorption of linearly polarized X-rays with the electric field vector pointing parallel or perpendicular to the MoS$_{2}$ sheet orientation, respectively^24,25^. We note that even though the 2H and 1T phase differ in the local microscopic structure, the S2s $\rightarrow$ S3p dipole transition generates equivalent in-plane S3p$_\parallel$ and out-of-plane S3p$_\perp$ orbital populations in both phases.

**Figure 2:**
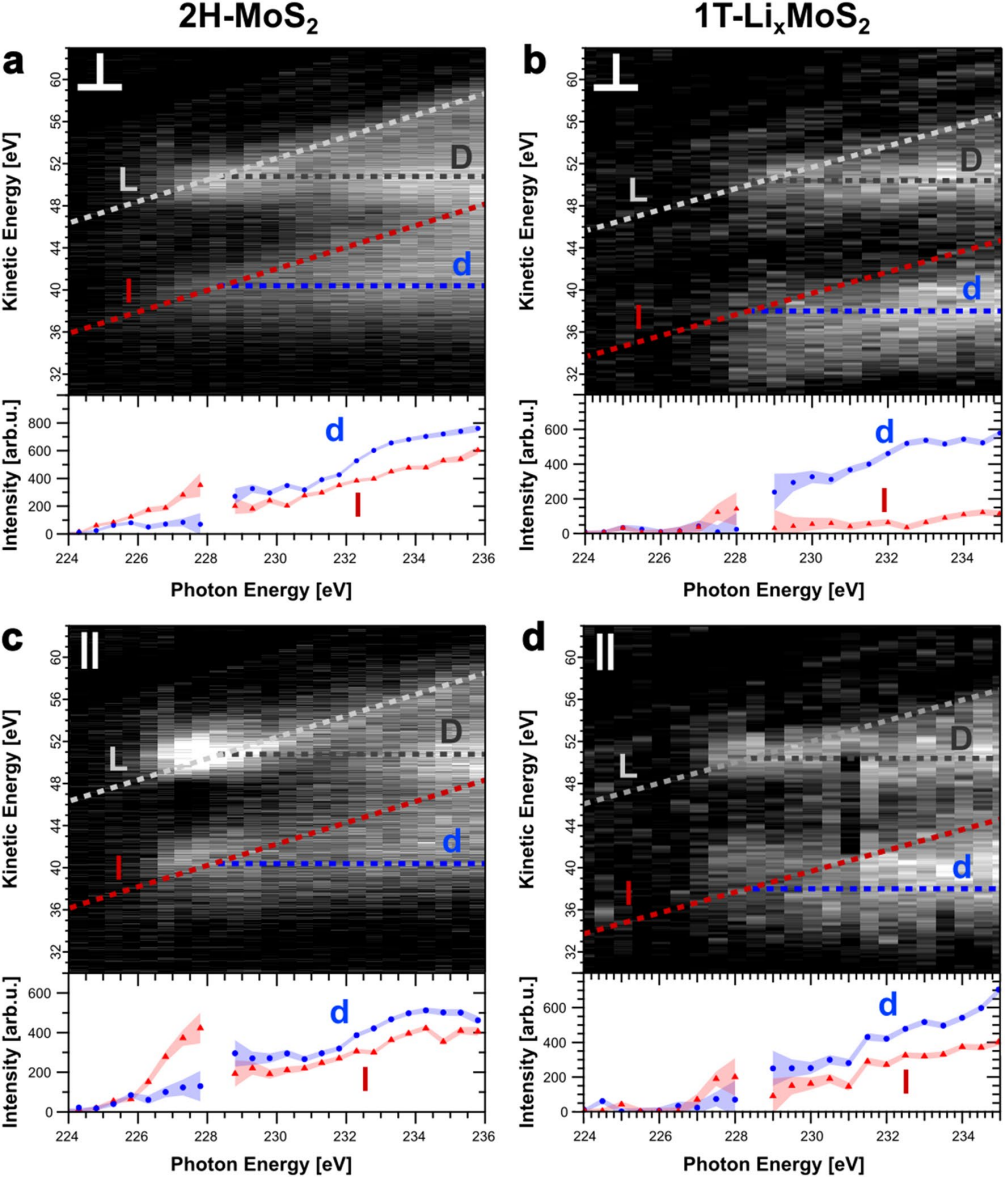
Polarization dependent S L$_{1}$L$_{2,3}$M$_{1,2,3}$ CK autoionization spectra as a function of incident X-ray energy for 2H-MoS$_{2}$ (**a**,**c**) and 1T-Li$_{\mathrm{x}}$MoS$_{2}$ (**b**,**d**). The Raman-channels l (S2p$^{-1}$3s$^{-1}$3p$^{1}$) and L (S2p$^{-1}$3p$^{-1}$3p$^{1}$) as well as the Auger-channels d (S2p$^{-1}$3s$^{-1}$deloc.$^{1}$) and D (S2p$^{-1}$3p$^{-1}$deloc.$^{1}$) are indicated. The symbols $\parallel$ and $\perp$ denote the selective preparation of in- and out-of-plane S3p excited states. The region of the direct S2p photoionization lines has been subtracted. Below each autoionization spectrum, the spectral intensities of the l- and d-channels are plotted as a function of the photon energy. Error bars represent the uncertainty of each individual fit.

Figure  2 shows polarization dependent S L$_{1}$L$_{2,3}$M$_{1,2,3}$ Coster-Kronig (CK) autoionization spectra, for 2H-MoS$_{2}$ (Fig. 2a,c) and 1T-Li$_{\mathrm{x}}$MoS$_{2}$ (Fig. 2b,d) as a function of incident photon energy. The photon energy has been tuned for in-plane and out-of-plane polarization across the S L$_{1}$ absorption edge from 224 eV to 236 eV. The autoionization electron spectra from the S L$_{1}$L$_{2,3}$M$_{1,2,3}$ CK decay are monitored in the kinetic energy range from 30 eV to 65 eV. In order to quantitatively evaluate the CK autoionization decay channels, all spectral features corresponding to direct S2p photoionization, shake-up and the secondary electron background have been subtracted according to a previously reported procedure^24,25^. A detailed description of the data analysis is given in the supporting information. Depending on the final state reached in the CK decay and based on their characteristic dispersive behaviour with the incoming X-ray photon energy, competing decay channels can be distinguished. For the Raman-channels S2p$^{-1}$3s$^{-1}$3p$^{1}$ (l) and S2p$^{-1}$3p$^{-1}$3p$^{1}$ (L) at constant binding energy the resonantly excited electron remains localized at the atomic site within the core hole lifetime. In contrast, the Auger-channels S2p$^{-1}$3s$^{-1}$deloc.$^{1}$ (d) and S2p$^{-1}$3p$^{-1}$deloc.$^{1}$ (D) appear at constant kinetic energy, which is the signature of S3p electron delocalization within the conduction band during the timescale of the S2s core hole decay, i.e. charge transfer occurred. At the resonance maximum of the S L$_{1}$-edge at a photon energy $h\nu _{res} = 228.5 \pm 0.1$ eV, the Raman-channels branch into charge transfer Auger-channels for both 2H-MoS$_{2}$ and 1T-Li$_{\mathrm{x}}$MoS$_{2}$. For 2H-MoS$_{2}$ the d- and D-channel are found at a kinetic energy of 40.4 eV (d) and 50.8 eV (D) while for 1T-Li$_{\mathrm{x}}$MoS$_{2}$ they are found at 38 eV (d) and 50.4 eV (D), respectively.

The quantitative analysis of the autoionization branching ratios and the related charge transfer times is based on the l- and d-channels, as these are spectrally pure autoionization final states^24^. As a function of incident photon energy, the intensities of the l- and d-channels are plotted in the lower panel below each resonant photoemission spectrum in Fig. 2. At and above resonance, both the localized Raman-channel (l) and delocalized Auger-channel (d) coexist with intensities $I_{Raman}$ and $I_{Auger}$. Below resonance only the Raman-channel is observable. With the CHC rate model $\tau _{CT}=I_{Raman}/I_{Auger} \cdot \tau _{S2s}$ using the natural S2s core hole lifetime $\tau _{S2s} = 0.51$ fs^39^, the timescale for charge delocalization can be determined in a range spanning $0.051 \,\text {fs}< \tau _{CT} < 5.1 \,\text {fs}$^24^. Following this procedure, a quantitative evaluation of the in- and out-of-plane charge transfer times $\tau _{CT}$ has been performed for 2H-MoS$_{2}$ and 1T-Li$_{\mathrm{x}}$MoS$_{2}$.

**Figure 3:**
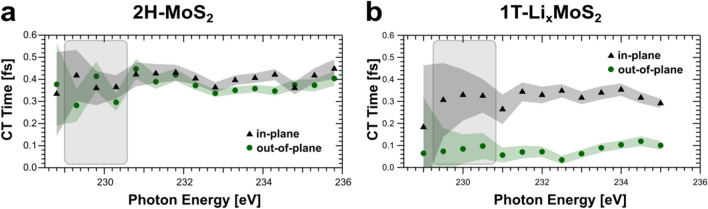
In-plane $\tau _{\parallel }$ (black triangles) and out-of-plane $\tau _{\perp }$ (green circles) charge transfer times of S3p excited states in 2H-MoS$_{2}$ (**a**) and 1T-Li$_{\mathrm{x}}$MoS$_{2}$ (**b**) from CHC analysis of Auger- (d) and Raman-channels (l) and the natural S2s core hole lifetime. Resonant excitation into the conduction band minimum occurs for incident photon energies 229.5 eV to 230.5 eV just above the S2s $\rightarrow$ S3p X-ray absorption resonance maximum. Error bars (shaded) for each photon energy, orientation and sample result from error propagation through the CHC analysis from the fitted intensities (Fig. 2) of each underlying autoionization spectrum.

Figure 3 summarizes the extracted charge transfer times of S3p$_\parallel$ and S3p$_\perp$ excited states as a function of the X-ray photon energy for 2H-MoS$_{2}$ (Fig. 3a) and 1T-Li$_{\mathrm{x}}$MoS$_{2}$ (Fig. 3b) based on the spectral intensities from Fig. 2. A slight variation of charge transfer above threshold excitation can be seen. The evaluation of the charge transfer times will be restricted to the photon energy range from 229.5 eV to 230.5 eV just above the S2s $\rightarrow$ S3p X-ray absorption resonance maximum (shaded in Fig. 3).

Semiconducting 2H-MoS$_{2}$ has S3p charge transfer times of $\tau _{2H,\parallel } = 0.38 \pm 0.08$ fs in-plane and $\tau _{2H,\perp } = 0.33 \pm 0.06$ fs out-of-plane. Metallic 1T-Li$_{\mathrm{x}}$MoS$_{2}$ has S3p charge transfer times of $\tau _{1T,\parallel } = 0.32 \pm 0.12$ fs in-plane and $\tau _{1T,\perp } = 0.09 \pm 0.07$ fs out-of-plane. By and large, the charge transfer in metallic 1T-Li$_{\mathrm{x}}$MoS$_{2}$ is for all directions faster in comparison to the semiconducting situation of 2H-MoS$_{2}$.

We start our discussion with the charge transfer dynamics in 2H-MoS$_{2}$. It is apparent from Fig. 3a that there is no significant difference between the in-plane and out-of-plane charge transfer times within the error margin. At first sight, this isotropic charge transfer seems puzzling, regarding the quasi-2D character displayed macroscopically by layered 2H-MoS$_{2}$. However, this result is explained, if we consider the local hexagonal arrangement of the S atoms around the Mo atoms within each S-Mo-S sheet as depicted in Fig. 1b: both the S2s $\rightarrow$ S3p$_\parallel$ and S2s $\rightarrow$ S3p$_\perp$ excitation populates orbitals, which have similar projections onto the direction of the angled covalent Mo-S bonds. Consequently equivalent S3p$_\parallel$ and S3p$_\perp$ charge transfer time constants are measured. This quasi-isotropic charge transfer in 2H-MoS$_{2}$ has been brought to attention previously by hard X-ray S K-edge CHC studies derived from systematically varied layer structures of MoS$_{2}$^30^. It has been proposed that the weak vdW interaction between adjacent MoS$_{2}$ layers is negligible^30^, and that intralayer electronic coupling must be the dominant delocalization pathway, which is in agreement with our microscopic explanation based on linear superposition of angled Mo-S bonds and the resulting isotropic S3p$_\parallel$ and S3p$_\perp$ charge transfer.

In contrast to the isotropic charge transfer in semiconducting 2H-MoS$_{2}$, highly anisotropic and faster charge transfer characteristics are found for metallic 1T-Li$_{\mathrm{x}}$MoS$_{2}$. Charge transfer from in-plane S3p$_\parallel$ orbitals within 1T-Li$_{\mathrm{x}}$MoS$_{2}$ accelerates by a factor of $\sim 1.2$ compared to 2H-MoS$_{2}$. As depicted in Fig. 1, electron injection upon lithiation into the Mo-S bonds is linked to a structural shift equivalent to a $60^\circ$ rotation of one full S atom plane. Thus, this change of occupancy in the Mo-S covalent bonds with concomitant structural changes is sensitively reflected by the in-plane S3p$_\parallel$ charge transfer acceleration.

The out-of-plane charge transfer in 1T-Li$_{\mathrm{x}}$MoS$_{2}$ takes place on a significantly shorter timescale, corresponding to an acceleration by a factor of $\sim 3.7$ compared to 2H-MoS$_{2}$. The value of $\tau _{1T,\perp }$ should be considered as an upper limit for the out-of-plane charge transfer time for the following reasons: our experimental geometry of $15^\circ$ grazing incidence adds a geometrical fraction of the slower in-plane charge transfer time to the faster out-of-plane component and our 1T-Li$_{\mathrm{x}}$MoS$_{2}$ crystal preparation with an average 1T phase concentration of 86 ± 5 % in the detection volume admixes some slower components from the coexisting 2H phase.

The key question is whether the significant acceleration of the out-of-plane charge transfer can be linked to the electronically and structurally changed properties of the semiconducting 2H-MoS$_{2}$ and metallic 1T-Li$_{\mathrm{x}}$MoS$_{2}$. As the distance between the vdW coupled S-Mo-S sheets increases in response to the lithiation and phase transition, no additional overlap between S orbitals in neighboring S-Mo-S sheets can be the root cause^6,12^. In addition the electron injection upon lithiation and the concomitant $60^\circ$ rotation of one full S atom plane changes the composition of the Mo-S angled bond for the out-of-plane and the in-plane projection in a similar fashion. Thus, the electron sharing between adsorbed/intercalated Li atoms with the S atoms of the S-Mo-S sheets seems to be the dominant process driving the accelerated S3p$_\perp$ charge transfer in 1T-Li$_{\mathrm{x}}$MoS$_{2}$. This explanation is substantiated by a comparison with adsorbed P3HT molecules on MoS$_{2}$ in the form of a P3HT/MoS$_{2}$/SiO$_{2}$ heterojunction^29^. The referred study showed that deposition of the conducting P3HT polymer molecules onto the MoS$_{2}$ monolayer leads to an n-doping (electron injection from the molecular donor into the MoS$_{2}$ film) accompanied by accelerated out-of-plane charge transfer of $\sim 50$ %, whereas the in-plane charge transfer is unaffected. In this system, overlap between the surface adsorbed P3HT frontier orbitals and S3p states can be the only cause for an efficient out-of-plane electron delocalization pathway, in particular since the other side of the heterojunction has insulating SiO$_{2}$ as a substrate. In analogy to the case of the P3HT/MoS$_{2}$/SiO$_{2}$ heterojunction, we conclude that the acceleration of $\tau _{1T,\perp }$ by a factor of $\sim 3.7$ compared to $\tau _{2H,\perp }$ in bulk lithiated, metallic 1T-Li$_{\mathrm{x}}$MoS$_{2}$ should also reflect the opening of an additional electron delocalization pathway due to the coupling of the S atoms of the S-Mo-S sheet to the adsorbed/intercalated Li.

With respect to the implementation of MoS$_{2}$ in energy storage devices like LIBs and supercapacitors, the results demonstrate that the quasi 2D layered structure, which enables efficient Li intercalation, does not restrict the electron mobility within the layer. In response to the Li intercalation reaction, the charge delocalization times reveal that the charge mobility is increased in both the in- and out-of-plane direction, facilitating charge extraction/insertion independent of the relative electrode orientation to the MoS$_{2}$ layers. Further, the results indicate that deposition/intercalation of atoms or molecules has the potential to greatly alter the out-of-plane charge transport properties of MoS$_{2}$. For instance, the catalytic performance of MoS$_{2}$ for hydrogen production has been shown to be hindered for catalytic active sites being separated from the conductive substrate by parallel oriented MoS$_{2}$ layers^40^. On this basis, deposition/intercalation could be used to modify out-of-plane charge mobility in MoS$_{2}$ to improve the properties of MoS$_{2}$ as a material for applications based on the out-of-plane charge mobility or where functionality is hindered by the interlayer potential barrier.

## Methods

### Core hole clock method

The principle of the CHC method is based on the resonant excitation of a core electron to an unoccupied state while the finite lifetime of the created core hole serves as an internal reference clock for the decay processes following the initial excitation in the studied system. Following the resonant excitation, competing decay channels of the autoionization process with different dispersive behavior can be observed. A linear dispersion between the X-ray energy and the kinetic energy of the electron is observed for resonantly excited electrons remaining localized within the core hole lifetime (Raman-channel). If the resonantly excited electron is delocalized within the conduction band during the decay process, i.e. charge transfer occurred, the kinetic energy of the ejected electron is independent of the exciting X-ray energy (Auger-channel). Scanning the X-ray energy across the resonance maximum, allows to unambiguously distinguish the different decay channels. The intensity ratio of the competing channels and the respective natural lifetime of the core-excited state are then used to calculate the charge transfer times of the studied system.

### XPS measurements

All experimental data presented in this work have been acquired, at the “FEMTOSPEX Molecules and Surfaces” endstation^41^, installed at the BESSY II soft X-ray UE56/1 PGM beamline during the single bunch operation mode. The beamline provides adjustable X-ray polarizations (linear vertical and horizontal as well as circular) with a focus spot size on the sample of approximately 200 x 200 ${\upmu }\mathrm{m}^{2}$. Absolute energy calibration was obtained by an Argon gas cell installed in the beamline. The data have been collected under ambient temperature and a base pressure in the measurement chamber of $2 \cdot 10^{-10}$ mbar. The presented data have been measured under a grazing X-ray incidence angle of $15^\circ$. A VG Scienta-Omicron angle-resolved time of flight (ARTOF) electron spectrometer with a $60^{\circ }$ acceptance angle lens system was used^42^. Further, preliminary measurements have been conducted at the LowDosePES endstation at the BESSY II PM4 dipole beamline.

### Sample preparation

Commercial 2H-MoS$_{2}$ crystals produced by 2D Semiconductors were cleaved by mechanical exfoliation under UHV conditions at a pressure of $< 10^{-9}$ mbar to ensure a pristine surface and were further annealed for 30 minutes at $460^\circ$C. The sample quality was verified by survey XPS spectra, showing no evidence of elements other than Mo and S.

A SAES Getters Li disperser was mounted at the chamber to achieve a controlled Li deposition directly onto the clean 2H-MoS$_{2}$ crystal surface. The total Li deposition time amounts to 80 minutes with a background pressure of $\sim 1 \cdot 10^{-8}$ mbar to successfully promote the transition to metallic 1T-Li$_{\mathrm{x}}$MoS$_{2}$.

## Supplementary Information

Supplementary Information
