# Supplementary material for: Directional charge delocalization dynamics in semiconducting 2H-MoS2 and metallic 1T-LixMoS2
Source: Sci Rep. 2021 Mar 25;11:6893. doi: 10.1038/s41598-021-86364-2 (PMC7994912; doi:10.1038/s41598-021-86364-2)
Supplement: Supplementary file 1 — Supplementary Information [file 41598_2021_86364_MOESM1_ESM.pdf]

Supplementary Information

Directional charge delocalization dynamics in semiconducting 2H-MoS_2 and metallic 1T-Li_xMoS_2

Robert Haverkamp [1,2,*], Nomi L. A. N. Sorgenfrei [1], Erika Giangrisostomi [1], Stefan Neppl [1], Danilo Kühn [1], Alexander Föhlisch [1,2]

[1] Helmholtz-Zentrum Berlin für Materialien und Energie GmbH, Methods and Instrumentation for Synchrotron Radiation Research PS-ISRR, Albert-Einstein-Straße 15 Berlin, 12489, Germany
[2] Institut für Physik und Astronomie, Universität Potsdam, Karl-Liebknecht-Straße 24/25 Potsdam, 14476, Germany

[*] Corresponding author e-mail: Robert.Haverkamp@Helmholtz-Berlin.de

XPS analysis of the Li deposition induced phase transition

After 10, 20, 50 and 80 minutes of Li deposition, a set of XPS spectra consisting of Valence band (VB) scans as well as Mo3d and S2p core level spectra have been recorded. The set of XPS spectra prior to Li deposition and after the final Li deposition step are shown in Supplementary Fig. 1. All binding energies (E_B) are referred to the Fermi level, determined from Au VB reference measurements (E_B = 0 eV at E_F). The Mo3$d_{5/2}$ and S2$p_{3/2}$ core level E_B are found at 229.71 eV and 162.45 eV, respectively with a spin-orbit splitting of 3.14 eV and 1.16 eV. After subtraction of a Shirley background, Gaussian fits with identical FWHM of 0.57 eV (S2p) and 0.75 eV (Mo3d) have been used, with intensity ratios of 1:2 for S2$p_{1/2}$:S2$p_{3/2}$ and 2:3 for Mo3$d_{3/2}$:Mo3$d_{5/2}$.

After 10 minutes of Li deposition, a $\sim$ 0.4 eV shift of the Mo3d and S2p core levels to higher E_B is observed. Upon further Li deposition, an additional pair of peaks emerges that with further deposition time became more intense than the initial Mo3d and S2p levels. In agreement with other studies these new peaks are shifted by about 0.8 ± 0.1 eV to lower E_B with respect to the initial peaks and are attributed to the 1T phase[1]. An additional pair of Gaussian peaks with identical spin-orbit splitting and intensity ratio as the corresponding core levels of 2H-MoS_2 have been used to model these components. It is found for both the 2H-MoS_2 S2p and Mo3d core levels that their FWHM only slightly increased by $\sim$ 0.1 eV whereas the 1T-Li_xMoS_2 S2p and Mo3d core levels have a FWHM of 1.1 eV and 0.7 eV, respectively.

The quantitative evaluation of the XPS intensity ratio revealed a dominant 1T phase concentration of 76 ± 5 % after the final Li deposition step. By increasing the surface sensitivity of the measurement by reducing the X-ray incidence angle, a 1T phase concentration of 86 ± 5 % was detected. This suggests weaker intercalation of Li atoms into the MoS_2 bulk, and that the Li deposition drives the phase transition predominantly within the surface layer. A substantial spectral modification and shift of the VB towards E_F further indicates the successful electron donation of the Li atoms to the 2H-MoS_2 lattice and the transition to the metallic 1T phase[1].

It should be mentioned that neither 2H-MoS_2 nor 1T-Li_xMoS_2 show a signature of sulfur vacancies in the crystal. The XPS signature of the sulfur vacancy doublet, which is energetically below the Mo3d and S2p core levels of both the 2H and the 1T phase signature, would be manifested as a shoulder on the low binding energy side of the respective core levels[2,3]. This non-detectable amount of sulfur vacancies confirms the high quality of the sample. Further we could not detect any time-dependent changes in the XPS spectra after the last Li deposition step, demonstrating the stability of the metallic state.

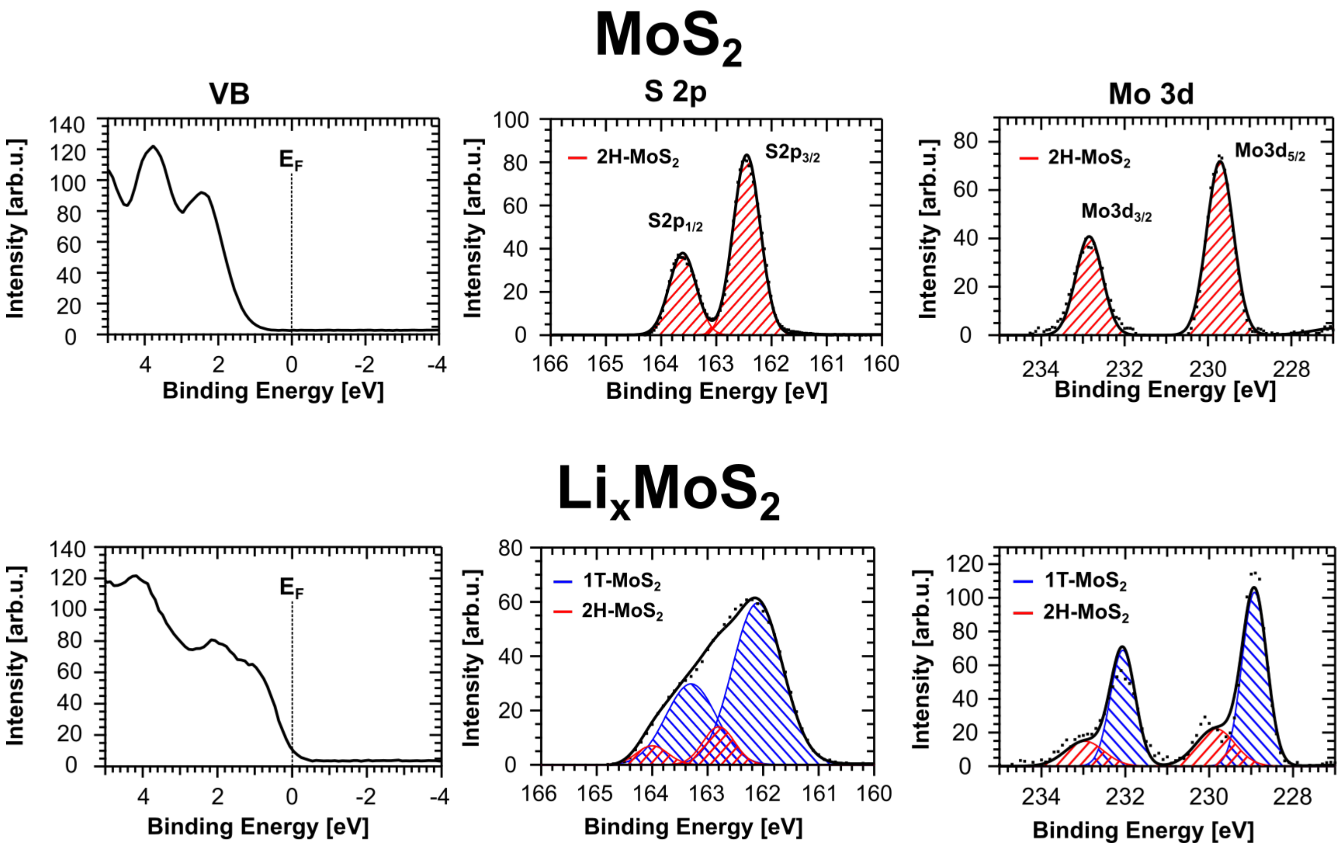

Supplementary Figure 1. XPS spectra of the VB as well as the S2p and Mo3d core levels prior to any Li deposition (upper panel) and after Li deposition (lower panel), confirming the successful transition from the 2H to the 1T phase. Contributions from the 1T and the 2H phase are indicated by blue and red hatched curves in the core level spectra.

Data analysis and spectral decomposition

Initially, all spectral intensities of the raw data were normalized to the X-ray photon flux of the incident synchrotron radiation. Since well below the S L_1-edge no resonant excitation occurs, the measured spectrum at this photon energy (E_{ph}) is exclusively composed of the features corresponding to the direct S2p photoionization and the secondary electron background. The secondary electron background is modeled by an exponentially decaying function $I_B(E_{kin}) = y_0 \cdot [1 + A^{-(\alpha \cdot E_{kin})}]$, where the best overall result is obtained for $\alpha = 0.06$ and A = 2.4. The amplitude parameter y_0 has been varied for each photon energy according to the spectral intensity at the electron kinetic energy $E_{kin} = 30$ eV. Subsequently, the determined lineshape of the S2p XPS main line, including the accompanying shake-up and shake-off features, has been shifted according to the varying X-ray energy and subtracted from each spectrum. For E_{ph} above the S L_1-edge, and after the subtraction of the secondary electron background as well as the S2p XPS main line and shake features, only the spectral components corresponding to the S $L_1L_{2,3}M_{1,2,3}$ CK autoionization decay following the S2s $\rightarrow$ S3p resonant excitation remain.
The spectral decomposition is exemplarily shown in the Supplementary Fig. 2 for 2H-MoS_2 (Supplementary Fig. 2a,c) and 1T-Li_xMoS_2 (Supplementary Fig. 2b,d) well below the S L_1-edge respectively at $E_{ph} = 225.8$ eV and $E_{ph} = 226.5$ eV and above the S L_1-edge respectively at $E_{ph} = 230.3$ eV and $E_{ph} = 231$ eV.

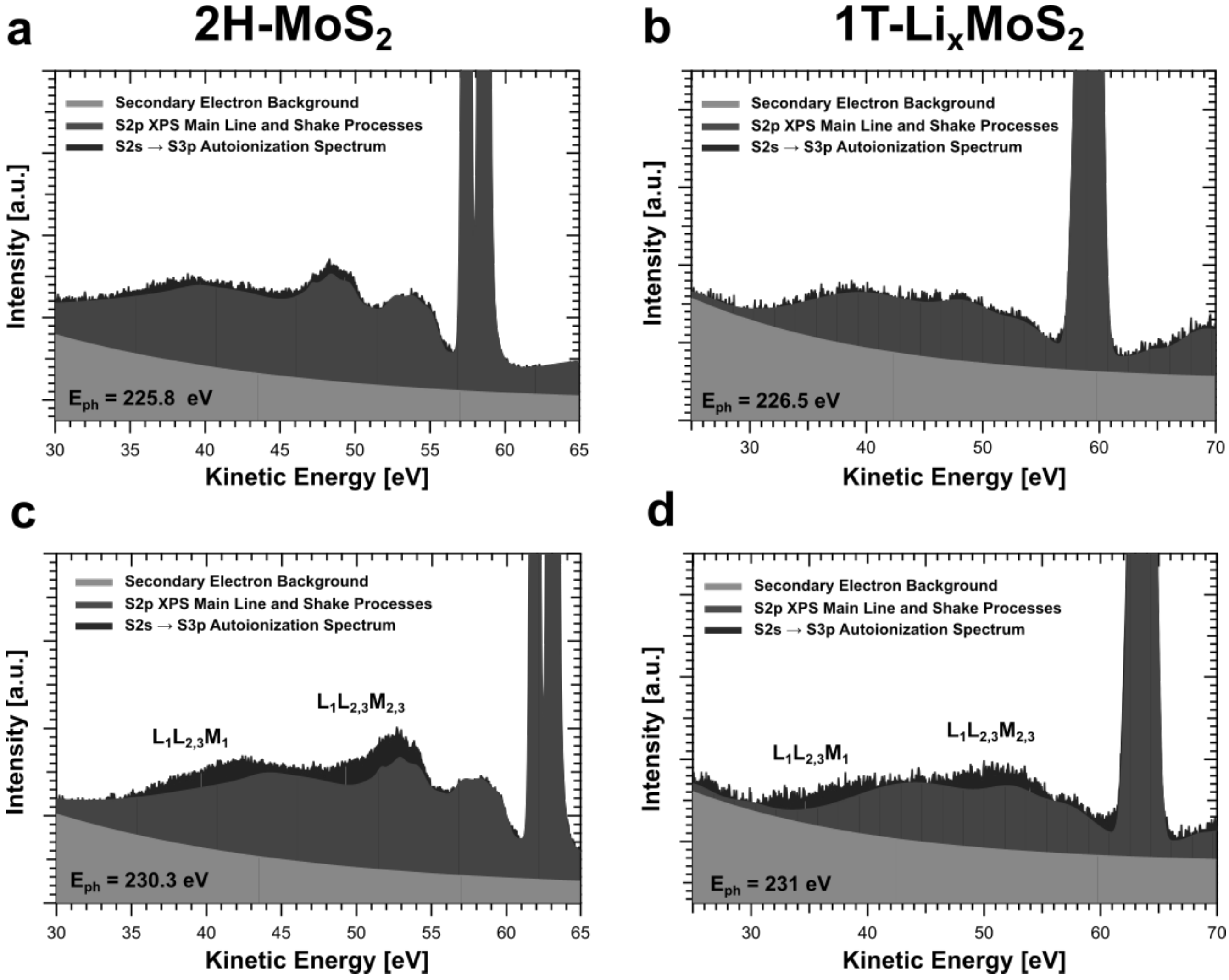

Supplementary Figure 2. Spectral decomposition and evolution for 2H-MoS_2 (**a** and **c**) and 1T-Li_xMoS_2 (**b** and **d**) exemplarily shown for exciting X-ray energies below (225.8 eV for 2H-MoS_2 and 226.5 eV for 1T-Li_xMoS_2) and above the S L_1-edge (230.3 eV for 2H-MoS_2 and 231 eV for 1T-Li_xMoS_2).

Quantitative CK autoionization decay channel analysis

To quantify the contributions of the Raman- and the Auger channels to the autoionization spectrum, a peak fitting routine has been used. In line with their microscopic origin, the charge transfer channels d and D (Auger- channels) have been fixed at constant E_{kin}, while the localized channels l and L (Raman- channels) are constrained to have constant E_B. Considering the experimental resolution and data quality, the spectral distribution of each decay channel is approximated by a Gaussian peak. The width of all four spectral features (d, D, l, L) was kept identical, while only their intensities are free parameters. For 2H-MoS_2, a FWHM of 5 eV was used, while for 1T-Li_xMoS_2 a slightly increased FWHM of 5.8 eV for all decay channel features resulted in the best overall fit. Exemplarily, the fitting procedure is shown in Supplementary Fig. 3 for 2H-MoS_2 (Supplementary Fig. 3a,c) at $E_{ph} = 230.3$ eV, as well as for 1T-Li_xMoS_2 (Supplementary Fig. 3b,d) at $E_{ph} = 230.5$ eV for horizontal ($\perp$) and vertical ($\|$) polarized X-rays, respectively.

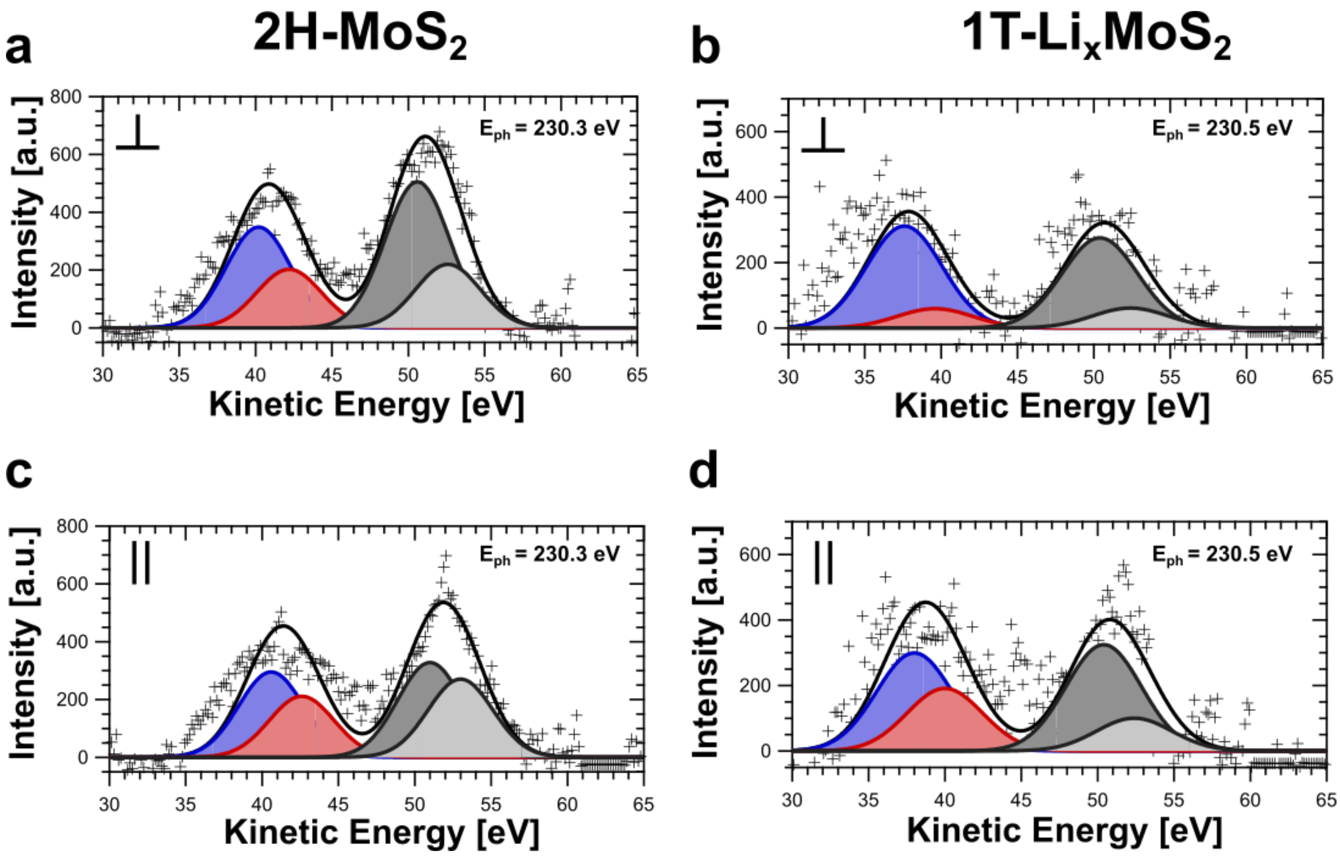

Supplementary Figure 3. Applied fitting routine to the polarization dependent $L_1L_{2,3}M_{1,2,3}$ autoionization spectra exemplarily shown for specific photon energies for 2H-MoS_2 and 1T-Li_xMoS_2. $\perp$ and $\|$ indicate the X-ray polarization relative to the MoS_2 crystal surface, promoting excitation into out-of-plane and in-plane S3p orbitals. Blue and red peaks depict the d- and the l-channels respectively, while the dark-grey and light grey peaks depict the D- and the L-channels. The polarization dependent autoionization spectra of 2H-MoS_2 at $E_{ph} = 230.3$ eV (**a** and **c**) and of 1T-Li_xMoS_2 at $E_{ph} = 230.5$ eV (**b** and **d**) are shown.

References

1. Papageorgopoulos, C. A. & Jaegermann, W. Li intercalation across and along the van der Waals surfaces of MoS_2(0001). *Surf. Sci.* **338**, 83–93, DOI: 10.1016/0039-6028(95)00544-7 (1995).

2. Zhang, X. *et al.* Unravelling the effect of sulfur vacancies on the electronic structure of the MoS_2 crystal. *Phys. Chem. Chem. Phys.* **22**, 21776–21783, DOI: 10.1039/c9cp07004d (2020).

3. Donarelli, M., Bisti, F., Perrozzi, F. & Ottaviano, L. Tunable sulfur desorption in exfoliated MoS_2 by means of thermal annealing in ultra-high vacuum. *Chem. Phys. Lett.* **588**, 198–202, DOI: 10.1016/j.cplett.2013.10.034 (2013).
